# Enhancing Relation Extraction for COVID-19 Vaccine Shot-Adverse Event Associations with Large Language Models

**DOI:** 10.21203/rs.3.rs-6201919/v1

**Published:** 2025-03-17

**Authors:** Yiming Li, Deepthi Viswaroopan, William He, Jianfu Li, Xu Zuo, Hua Xu, Cui Tao

**Affiliations:** The University of Texas Health Science Center at Houston; The University of Texas Health Science Center at Houston; Duke University; Mayo Clinic; The University of Texas Health Science Center at Houston; Yale University; Mayo Clinic

**Keywords:** Relation extraction, VAERS, Generative pre-trained transformer (GPT), Large language model (LLM), Social media, Deep learning

## Abstract

**Objective:**

The rapid evolution of the COVID-19 virus has led to the development of different vaccine shots, each designed to combat specific variants and enhance overall efficacy. While vaccines have been crucial in controlling the spread of the virus, they can also cause adverse events (AEs). Understanding these relationships is vital for vaccine safety monitoring and surveillance.

**Methods:**

In our study, we collected data from the Vaccine Adverse Event Reporting System (VAERS) and social media platforms (Twitter and Reddit) to extract relationships between COVID-19 vaccine shots and adverse events. The dataset comprised 771 relation pairs, enabling a comprehensive analysis of adverse event patterns. We employed state-of-the-art GPT models, including GPT-3.5 and GPT-4, alongside traditional models such as Recurrent Neural Networks (RNNs) and BioBERT, to extract these relationships. Additionally, we used two sets of post-processing rules to further refine the extracted relations. Evaluation metrics including precision, recall, and F1-score were used to assess the performance of our models in extracting these relationships accurately.

**Results:**

The most commonly reported AEs following the primary series of COVID-19 vaccines include arm soreness, fatigue, and headache, while the spectrum of AEs following boosters is more diverse. In relation extraction, fine-tuned GPT-3.5 with Sentence-based Relation Identification achieved the highest precision of 0.94 and a perfect recall of 1, resulting in an impressive F1 score of 0.97.

**Conclusion:**

This study advances biomedical informatics by showing how large language models and deep learning models can extract relationships between vaccine shots and adverse events from VAERS and social media. These findings improve vaccine safety monitoring and clinical practice by enhancing our understanding of post-vaccination symptoms. The study sets a precedent for future research in natural language processing and biomedical informatics, with potential applications in pharmacovigilance and clinical decision-making.

## INTRODUCTION

COVID-19, caused by the novel coronavirus SARS-CoV-2, emerged in late 2019 and quickly evolved into an international pandemic, posing significant threats to public health and economies worldwide [1], [2]. The rapid spread of the virus led to an urgent need for effective vaccines to mitigate its impact [3]. Vaccination campaigns have played a crucial role in controlling the spread of the virus and reducing the severity of illness, contributing significantly to global efforts to combat the epidemic [4], [5], [6]. Consequently, multiple vaccines were developed at an unprecedented pace, utilizing various technologies such as mRNA, viral vector, and protein subunit platforms [7]. These vaccines underwent rigorous testing in clinical trials to ensure safety and efficacy [8], [9]. Emergency Use Authorization (EUA) was granted by regulatory agencies based on promising trial results, leading to the widespread distribution and administration of COVID-19 vaccines [10].

While COVID-19 vaccines have been instrumental in controlling the spread of the virus, they can also cause adverse events (AEs) in some individuals [11], [12], [13], [14], [15], [16], [17], [18], [19]. These AEs are typically mild and transient, including pain at the injection site, fatigue, headache, muscle pain, chills, fever, and nausea [20]. However, in rare cases, more serious AEs such as allergic reactions, myocarditis, and blood clotting disorders have been reported [21]. The incidence of these serious AEs is extremely low, with rates ranging from 3 to 5 cases per million doses for myocarditis, and 3.83 cases per million doses for thrombosis with thrombocytopenia syndrome (TTS) [22], [23]. While these AEs are rare, they can have serious consequences, such as hospitalization or even death [24]. Additionally, AEs can contribute to vaccine hesitancy, leading to lower vaccination rates and hindering efforts to achieve herd immunity, which is critical for ending the pandemic [25].

Individuals are encouraged to report any adverse events following immunization (AEFI) to the Vaccine Adverse Event Reporting System (VAERS), a passive surveillance system co-managed by the Centers for Disease Control and Prevention (CDC) and the Food and Drug Administration (FDA) [26], [27]. VAERS collects and analyzes reports of AEFI to help identify potential safety concerns and ensure the continued safety of vaccines [28]. In addition to VAERS, individuals often share their experiences and concerns about AEFI on social media platforms such as Twitter and Reddit [29]. These platforms provide a valuable source of real-world data, offering insights into the public’s perception and experiences with COVID-19 vaccines [30]. As of April 2024, VAERS has received 970,670 reports of AEs following COVID-19 vaccination [31]. Additionally, there have been almost 300 million English-language Tweets and more than 266,840 posts on Reddit discussing COVID-19 vaccines, highlighting the significance of social media in capturing and understanding public sentiment and experiences related to vaccination [32], [33].

Relation extraction (RE) aims to identify and classify relationships between entities in text, such as the relationship between COVID-19 vaccine shots and AEs [34], [35]. He et al. focusing on the chemical-protein relation and drug-drug interaction, developed a prompt tuning model for biomedical relation extraction to enhance the performance of pre-trained language models in few-shot scenarios [36]. In recent years, deep learning has significantly advanced information extraction tasks by enabling models to automatically learn hierarchical features from large annotated datasets, improving accuracy and adaptability in various domains [37], [38]. Currently, Large Language Models (LLMs) have revolutionized natural language processing (NLP) by leveraging extremely massive amounts of text data to learn complex language patterns [39], [40], [41], [42]. These models, such as GPT (Generative Pre-trained Transformer) and BERT (Bidirectional Encoder Representations from Transformers), have demonstrated state-of-the-art performance in various NLP tasks, including relation extraction [11], [43], [44], [45], [46]. Wan et al. proposed GPT-RE to improve RE with LLMs like GPT-3 [47]. GPT-RE addresses LLMs’ shortcomings in RE by incorporating task-specific entity representations and enriching demonstrations with gold label-induced reasoning logic [47]. Evaluation on four RE datasets shows that GPT-RE outperforms both existing GPT-3 baselines and fully-supervised baselines, achieving state-of-the-art performances on Semeval and SciERC datasets, and competitive performances on TACRED and ACE05 datasets [47]. LLMs are particularly effective in relation extraction due to their ability to capture contextual information and semantic nuances in language [48]. However, there are few studies related to RE with fine-tuned LLMs. Li et al. compared the performance of Generative Pre-trained Transformers (GPT) with traditional models (LSTM and BioBERT) in extracting relations related to acupoint locations from textual sources [43]. They used the WHO Standard Acupuncture Point Locations corpus and annotated five types of relations between acupoints [43]. The results showed that fine-tuned GPT-3.5 outperformed other models, indicating the effectiveness of large language models in this context and their potential in advancing informatics applications in traditional and complementary medicine [43]. By fine-tuning LLMs on annotated datasets, researchers can train models to accurately extract relations from text, enabling a wide range of applications in biomedical informatics and beyond [49].

The objective of this study is to investigate the use of LLMs and deep learning models for extracting relations between different shots of COVID-19 vaccines and the AEs caused by them. This research is significant as it addresses the need for efficient methods to extract valuable insights from textual data sources such as VAERS reports, Tweets, and Reddit posts. By leveraging the capabilities of LLMs and deep learning models, we aim to improve the accuracy and efficiency of relation extraction, contributing to a better understanding of the relationships between vaccine shots and AEs. This understanding is crucial for ensuring the safety and efficacy of COVID-19 vaccination programs, ultimately aiding in public health decision-making and enhancing vaccine safety surveillance systems.

## METHODS

**Figure 1:**
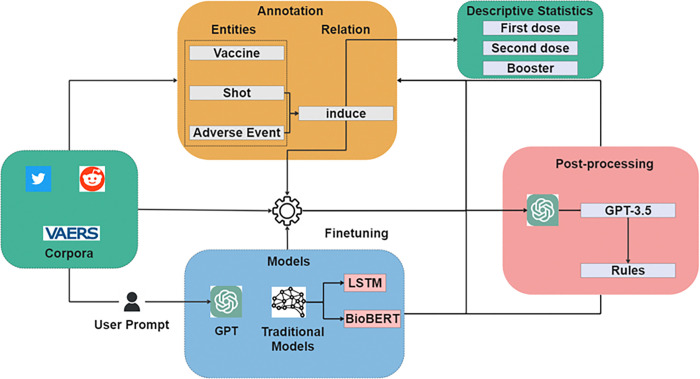
Overview of the Framework

Figure 1 provides an overview of our study, which focuses on analyzing AEs following COVID-19 vaccination. We collected data from VAERS and social media platforms (Twitter and Reddit) to extract relationships between vaccine shots and adverse events. Using state-of-the-art GPT models, we compared their performance in relation extraction with traditional models. Additionally, we counted these relationships to compare them with the results obtained from structured data. This comprehensive approach allows us to gain insights into AE patterns following COVID-19 vaccination and evaluate the effectiveness of different models in extracting relevant information from unstructured data sources.

### Data sources

In this study, we employed a multifaceted approach to extract and analyze the relationships between COVID-19 vaccine shots and AEs. In this study, we utilized the same two primary data sources as those used by Li et al.: VAERS and user-generated content from various social media platforms (Twitter, Reddit) [50]. The corpus included 230 VAERS reports, 3,383 tweets, and 49 Reddit posts. This dataset allowed us to explore a wide range of AEs reported by vaccine recipients.

### VAERS

VAERS is a crucial surveillance program co-managed by the Centers for CDC and FDA in the United States [51, pp. 2006–2015]. Established in 1990, VAERS serves as a vital tool for monitoring the safety of vaccines licensed for use in the country [52]. It operates as a passive reporting system, meaning that healthcare providers, vaccine manufacturers, and the public can submit reports of AEs that occur after vaccination [53]. VAERS collects and analyzes these reports to detect any unusual or unexpected patterns of AEs, which helps to identify potential vaccine safety concerns and ensure the continued safety of vaccines [54].

#### Twitter

Twitter, a prominent social media platform, has emerged as a valuable source of real-time information and communication during the COVID-19 pandemic [55]. With millions of users worldwide, Twitter provides a platform for individuals to share their experiences and concerns related to various aspects of the pandemic, including vaccination, at an approximate rate of reporting a COVID-19 related tweet every 45 milliseconds [56]. Users often turn to Twitter to discuss their symptoms, side effects, and overall experiences after receiving the vaccine, creating a rich source of real-world data [57]. This data can offer insights into the public’s perception and experiences with COVID-19 vaccines, complementing traditional surveillance systems like VAERS.

#### Reddit

Reddit, a popular social media platform known for its diverse range of communities (subreddits), has become a hub for discussions related to COVID-19 and vaccination [58]. With its large and varied user base, Reddit provides a unique space for individuals to share their experiences, questions, and concerns regarding COVID-19 vaccines [59]. Understanding these perspectives is crucial for public health authorities and researchers seeking to address vaccine hesitancy and ensure the success of COVID-19 vaccination efforts.

### Annotation

In the annotation process, we invited two annotators to identify one type of relationship: *induce*. *Induce* refers to the relationship between the shot and the specific triggered AEs. The Identification of this relationship is crucial as it helps us understand the correlation link between vaccine shots and AEs, providing valuable insights into vaccine safety and potential risk factors.

**Figure 2:**
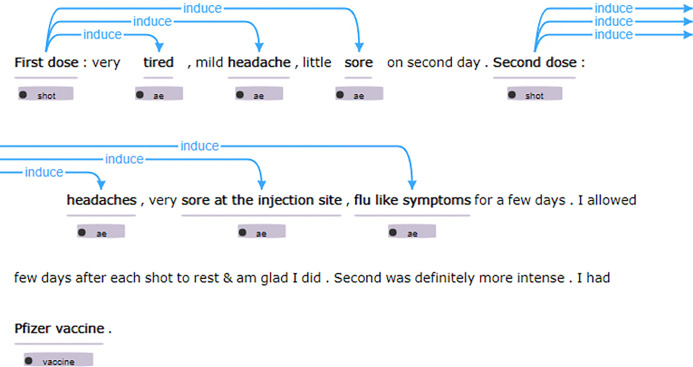
An annotation example in this study

Figure 2 provides an example of annotated relationships in this study. In the provided text, the administration of the first dose of the vaccine is associated with symptoms such as feeling very tired, experiencing a mild headache, and having slight soreness on the second day following vaccination. These symptoms collectively construct an *induce* relationship, indicating an association between the vaccine shot and the reported AEs. Similarly, the text describes the administration of the second dose, which results in headaches, significant soreness at the injection site, and flu-like symptoms lasting for a few days. These symptoms also form an ‘induce’ relationship, suggesting a potential causal association between the second vaccine dose and the reported adverse events.

### Models

In this study, we used advanced language models and deep learning techniques, including GPT, Recurrent Neural Networks (RNNs), and a pre-trained biomedical language representation model for biomedical text mining (BioBERT), to extract relationships between different shots of COVID-19 vaccines and the adverse events they cause.

### GPT

GPT is a state-of-the-art language model known for its ability to understand and generate human-like text [4], [9], [37], [50], [60]. Developed by OpenAI, GPT has gained popularity in the field of RE due to its remarkable performance and versatility [35]. One of the key strengths of GPT lies in its pre-training on a vast amount of text data, which enables it to learn complex language patterns and relationships [60]. This pre-training allows GPT to effectively capture the contextual information necessary for relation extraction tasks, making it particularly well-suited for analyzing unstructured text data, such as medical reports or social media posts [40]. Additionally, GPT’s transformer architecture allows it to process long-range dependencies in text, which is essential for identifying relationships between entities that may be distant from each other in a sentence or document [61].

### RNN

RNNs are a class of neural networks particularly well-suited for sequential data, making them valuable for tasks such as RE [62]. Unlike traditional feedforward neural networks, RNNs have connections that form a directed cycle, allowing them to maintain a state or memory of previous inputs [63]. This sequential nature enables RNNs to effectively capture the context and dependencies between words in a sentence, which is essential for extracting relationships between entities in text [64]. In RE tasks, RNNs can be used to analyze the sequential structure of sentences and identify patterns that indicate a specific relationship between entities, such as the relationship between COVID-19 vaccine shots and AEs [65].

#### BioBERT

BioBERT is a variant of the BERT (Bidirectional Encoder Representations from Transformers) model that has been specifically trained on biomedical text [66]. BioBERT’s training on biomedical data allows it to effectively identify and classify relationships between entities. Its specialized training makes BioBERT a valuable tool for extracting meaningful insights from biomedical text, aiding researchers in understanding complex relationships and phenomena in the biomedical domain.

### Experiment setup

#### Data Split

We reused the data split by Li et al., which included 2929 posts/reports for training, 365 posts/reports in the validation set, and 368 posts/reports in the test set. This split ensured a sufficient amount of data for training while maintaining separate sets for validation and testing.

### GPT

In this study, we employed pretrained GPT-3.5 (version: gpt-3.5-turbo-16k) and GPT-4 (version: gpt-4–32k). These models were selected for their advanced natural language processing capabilities and extensive training on diverse text corpora.

To adapt the models to the task of extracting relationships between vaccine shots and adverse events, we fine-tuned GPT-3.5 (version: gpt-3.5-turbo-16k) specifically for this purpose. The fine-tuning process involved updating the model’s weights using our annotated dataset to improve its performance on this specific task.

We used a temperature of 1.0 for all GPT models, except for the fine-tuned GPT-3.5, which used a temperature of 0.3. The maximum output token length for all models was set to 4,096, ensuring that the models could generate sufficient context for the task.

For the pretrained GPT-3.5 and GPT-4 models, we used the following prompts:

“system”:”You are a relation retrieval expert that is good at extrating relations between entities”

“user”: “The actual description text is here:’{text}’. We have a description for each adverse event report. We have annotated the entities. The entity type includes ‘vaccine’, ‘shot’, and ‘ae’(‘adverse events’). Now we have to conduct the relation extraction task. We want to extract ‘induce’ first. ‘induce’ refers to the shot associated with the adverse event. The starting entity type of ‘induce’ is ‘shot’, and the end entity type is ‘ae’. Entities({entities}) have been annotated. And each entity is formated as “‘Entity sequence number’’\t’’Entity type’’ ‘‘starting character location’’ ‘‘end character location’’\t’’Entity’“. To facilitate output extraction, please output the realtion directly and format the relation as “‘Relation sequence number(Starting from R1, R2,...)’’\t’’induce’’ ‘‘Arg1:Entity sequence number’’ ‘‘Arg2:Entity sequence number’’\n’“from the following text. If no relations, please do not output anything.”

For the fine-tuned GPT-3.5 model, we used the following prompt: ***.

“system”: “You are a relation retrieval expert that is good at extracting relations between entities. To facilitate output extraction, please output the relation directly and format the relation as Relation sequence number(Starting from R1, R2,...)\tdirection_of Arg1:Entity sequence number Arg2:Entity sequence number\nfrom the following text. If no relations, please do not output anything.”

“user”: “Text information is below.\n---------------------\n{txt}\n---------------------\nEntity information is below(Format is:’T(id)\t(entity_type) (start_offset) (end_offset)’\t’(entity_text)’){entities}\nQuery:Now we have to conduct the relation extraction task. We want to extract ‘induce’ rst. ‘induce’ refers to the shot associated with the adverse event. The starting entity type of ‘induce’ is ‘shot’ entity and the end entity type of ‘induce’ is the ‘ae’ entity.”,

These prompts were designed to guide the models in generating responses related to the relationship *induce* between the vaccine shots and AEs.

### LSTM

We fine-tuned an LSTM model for this study, utilizing a character-level architecture with specific parameters. These parameters include enabling lowercasing, setting the character embedding dimension to 25, the character LSTM hidden layer size to 25, the token embedding dimension to 200, the token LSTM hidden layer size to 100, and a dropout rate of 0.5.

For training, we used a batch size of 32 and a learning rate of 1e-3. The maximum number of training steps was set to 30,000. These parameters were carefully chosen to balance model complexity and training efficiency, aiming to achieve optimal performance in extracting relationships between vaccine shots and adverse events.

#### BioBERT

For our BioBERT model, we utilized BioBERT v1.1, fine-tuned specifically for the task of extracting relationships between vaccine shots and AEs. The maximum total input sequence length after WordPiece tokenization was set to 128. Sequences longer than this were truncated, while sequences shorter than this were padded to meet the required length.

We set the training batch size to 32 and the evaluation batch size to 8. The initial learning rate for the Adam optimizer was set to 5e-5, and the total number of training epochs was set to 3.0. Additionally, we performed a linear learning rate warmup for 10% of the training (warmup_proportion = 0.1).

We saved the model checkpoint every 1000 steps (save_checkpoints_steps = 1000) and made 1000 steps in each estimator call (iterations_per_loop = 1000). These settings ensured that the model was regularly saved and that training progress was appropriately logged for monitoring and analysis.

#### Post-processing

In our study, we aimed to further improve the performance of the fine-tuned GPT-3.5 model by employing and testing two sets of post-processing rules. These rules were designed to refine the extracted relations between vaccine shots and AEs.

#### Rule 1: Sentence-based Relation Identification

The first set of rules focused on identifying relations that occurred within the same sentence. This approach aimed to capture more direct and contextually relevant associations between shots and AEs. For each shot and AE pair, we extracted all relations that were mentioned within the same sentence. This method helped to ensure that the extracted relations were closely related in the text, enhancing the overall quality of the extracted data.

#### Rule 2: Character Offset-based Relation Identification

The second set of rules was based on character offset distances between shots and AEs. For each extracted shot, we identified the AE with the shortest distance in character offset as the related ae. This rule aimed to capture relations that were mentioned in close proximity within the text, assuming that closer mentions were more likely to be directly associated.

### Evaluation

For evaluation, we employed strict metrics including precision, recall, and F1-score to assess the performance of our models in identifying and linking vaccine shots with specific adverse events. Strict precision measures the proportion of correctly identified relationships (true positives) out of all identified relationships, ensuring that only exact matches are considered. Recall measures the proportion of correctly identified relationships out of all relationships that should have been identified. F1-score, the harmonic mean of precision and recall, provides a balanced measure of a model’s performance.

## RESULTS

The distribution of ‘induce’ relationships across the training, validation, and test sets is illustrated in Table 1. The training set contains 647 instances, which are used for model learning and parameter adjustment. The validation set includes 65 relationships, serving to optimize the model’s performance during development. The test set consists of 59 relationships, used to assess the model’s ability to generalize to unseen data. The inter-rater agreement for the annotation task is 0.6, indicating moderate agreement between the annotators.

**Figure 3:**
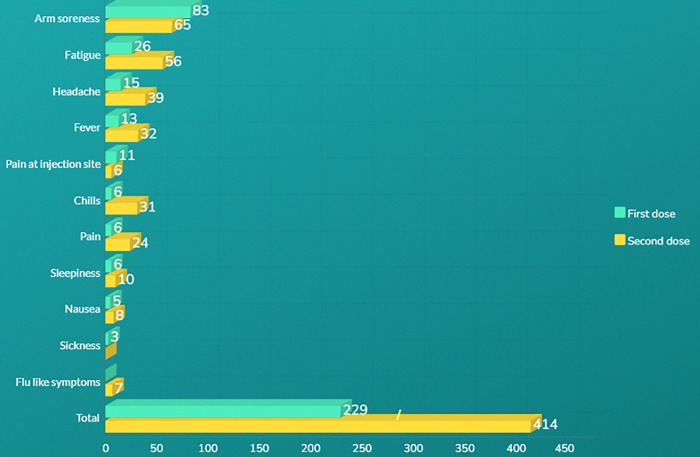
Total Count and Counts of Top 10 symptoms mentioned in the corpora for primary series of COVID-19 vaccine

Figure 3 provides a comparison of the total counts and the counts of the top 10 symptoms reported after the primary series of COVID-19 vaccines in the corpora. For the first dose, the most commonly reported symptoms were arm soreness (83 mentions), fatigue (26 mentions), and headache (15 mentions). Similarly, after the second dose, arm soreness was still the most frequently reported symptom (65 mentions), followed by fatigue (56 mentions) and headache (39 mentions).

**Figure 4:**
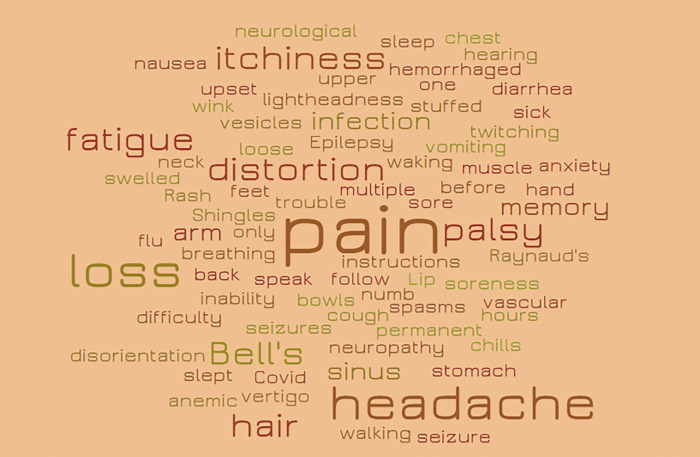
Word cloud for symptoms mentioned in the COVID-19 booster vaccine in the corpora

Figure 4 presents a word cloud depicting the diverse range of symptoms reported following COVID-19 booster doses mentioned in the corpora. The word cloud illustrates the frequency of various symptoms, with larger words indicating higher frequency. Symptoms such as pain (n = 7), headache (n = 5) and Bell’s palsy (n = 3) are among the most frequently reported. Other notable symptoms include hair loss, itchiness, and fatigue, each mentioned three times. The word cloud reflects the wide array of symptoms experienced by individuals after receiving a COVID-19 booster, ranging from mild discomforts such as soreness and chills to more severe conditions such as seizures and neurological issues.

**Figure 5:**
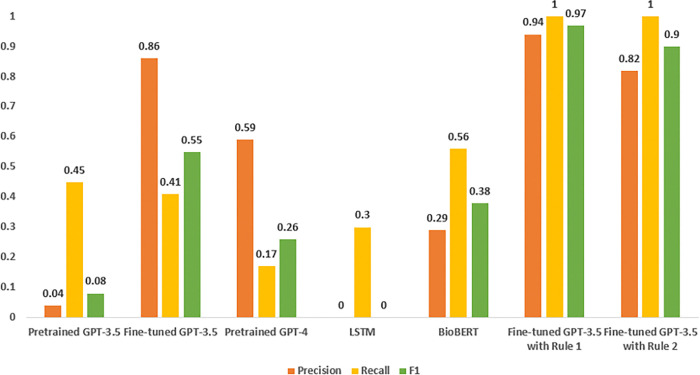
Performance (precision, recall, F1) across different models in “induce” relation extraction

Figure 5 provides an overview of the performance of different models in the extraction of the ‘induce’ relationship. The fine-tuned GPT-3.5 model demonstrated strong performance with a precision of 0.86, although its recall was slightly lower at 0.41, resulting in an F1 score of 0.55. Fine-tuned GPT-3.5 with rule-based post-processing (rule1) achieved the highest precision of 0.94 and a perfect recall of 1, leading to an impressive F1 score of 0.97. Another rule-based approach with GPT-3.5 (rule2) also performed well, with a precision of 0.82 and perfect recall, resulting in an F1 score of 0.90. In comparison, the pretrained GPT-4 model exhibited a precision of 0.59 and an F1 score of 0.26, indicating lower performance than the fine-tuned GPT-3.5 models. LSTM showed limitations with zero precision, indicating potential issues with false positives, and an F1 score of 0, highlighting challenges in accurately extracting the ‘induce’ relationship. BioBERT achieved a higher recall of 0.56 but with a lower precision of 0.29, leading to an F1 score of 0.38.

## DISCUSSION

The study presents a comprehensive analysis of adverse events following COVID-19 vaccination, focusing on the extraction of shot-adverse event relationships from self-reported data sources. By utilizing state-of-the-art language models and incorporating domain-specific rules, the study sheds light on the specific AEs associated with different shots of COVID-19 vaccines.

### Findings

The annotated data provided insights into specific AEs associated with different shots of COVID-19 vaccines. After the first dose, commonly reported symptoms included arm soreness, fatigue, and headache, while after the second dose, these symptoms persisted alongside arm soreness, fatigue, and headache. This finding aligns with Li et al.’s analysis of structured data on AEs following COVID-19 vaccines [15]. This consistency in the spectrum of reported AEs following the first two doses is crucial for understanding and managing post-vaccination symptoms. The similarity in AEs suggests a consistent pattern of immune response to the vaccine across both shots. One possible explanation is that the first dose primes the immune system, introducing it to the vaccine’s antigens and initiating an immune response [67]. The second dose then serves to reinforce and enhance the immune response initiated by the first dose [68]. This process leads to a more robust and comprehensive immune response, which may explain why the AEs reported after the second dose are often similar to those after the first dose. For COVID-19 booster doses, the analysis reveals a diverse range of symptoms, as depicted in Fig. 4. Symptoms such as pain, headache, and Bell’s palsy are among the most frequently reported, along with hair loss, itchiness, and fatigue. This diversity and severity of symptoms for COVID-19 boosters could be attributed to several factors. Firstly, booster doses are administered to individuals who have already received the primary series of COVID-19 vaccines, potentially leading to a different immune response compared to the initial shots [69], [70]. Additionally, the emergence of new variants of the virus may influence the immune response to booster doses, resulting in different AEs [71]. Furthermore, individuals receiving booster doses may have underlying health conditions or age-related factors that contribute to the diversity and severity of adverse events.

In this task, we evaluated and fine-tuned state-of-the-art models to extract relations between vaccine shots and AEs. The success of GPT-3.5 in relation extraction can be attributed to its ability to understand and generate natural language text, which is crucial for processing unstructured data like medical reports and social media posts [43]. GPT-3.5’s large model size and extensive training on diverse text data enable it to capture complex patterns and relationships in language, making it well-suited for tasks like relation extraction [43]. The new model, Llama 3, with its low token limit of 512, is not suitable for this relation extraction task, as it may not have the capacity to process the entire text of medical reports or social media posts, which can be lengthy and contain crucial information at various parts of the text.

Fine-tuning GPT-3.5 for relation extraction involves training the model on a dataset that includes examples of the relationships between vaccine shots and AEs. During this process, the model adjusts its parameters to better understand the nuances of the data, such as the language used to describe AEs and the context in which they occur in relation to vaccine shots. By learning from the annotated data, GPT-3.5 becomes more adept at identifying relevant information and extracting accurate relationships, resulting in improved precision and recall in relation extraction tasks related to vaccine safety monitoring.

Rule 1, the sentence-based relation Identification method, complements the fine-tuned GPT-3.5 model by providing a structured approach to identifying and extracting relations. This method takes advantage of the grammatical structure of sentences to identify key elements that indicate a relationship between vaccine shots and AEs. By focusing on the syntactic and semantic patterns within sentences, Rule 1 helps the model pinpoint relevant information more effectively, leading to a higher accuracy in relation extraction. Additionally, incorporating domain-specific rules ensures that the model considers context-specific factors that may influence the relationship between vaccine shots and AEs, further enhancing its ability to capture these relationships accurately.

In contrast, LSTM and BioBERT show limitations in relation extraction for this task. LSTM’s zero precision indicates potential issues with false positives, while BioBERT’s lower precision suggests that it may struggle with accurately extracting relations in this context. These findings emphasize the importance of choosing models and techniques that are well-suited for the specific requirements and complexities of biomedical tasks like relation extraction in vaccine safety monitoring.

### Strengths and limitations

This study employs a multifaceted approach, utilizing data from both structured sources like VAERS and unstructured sources like social media (Twitter and Reddit) to extract relationships between vaccine shots and adverse events. This comprehensive methodology provides a more holistic view of adverse events following COVID-19 vaccination. The study also utilizes advanced LLMs and deep learning techniques, including GPT and BioBERT, known for their effectiveness in natural language processing tasks. These models are specifically fine-tuned for the task of relation extraction, enhancing their ability to extract meaningful insights from unstructured text data. Additionally, the study introduces novel post-processing rules, particularly Rule 1 (Sentence-based Relation Identification), to further improve the performance of the fine-tuned GPT-3.5 model. These rules are designed to refine the extracted relations, ensuring that only relevant and contextually accurate relationships are considered.

While fine-tuning GPT-3.5 proves effective in this study, it is important to note that this process is task-specific and may not generalize well to other relation extraction tasks. Further studies are needed to evaluate the model’s performance in different contexts. Another limitation is the lack of generalizability, as the study focuses specifically on relation extraction between vaccine shots and AEs following COVID-19 vaccination. While the findings are valuable for vaccine safety monitoring, they may not be directly applicable to other domains or types of AEs.

## CONCLUSION

This study significantly advances biomedical informatics by demonstrating the effectiveness of state-of-the-art LLMs and deep learning techniques in extracting relationships between vaccine shots and AEs from diverse data sources, including VAERS and social media platforms. The findings enhance vaccine safety monitoring and AE surveillance, benefiting clinical practice by improving the understanding and management of post-vaccination symptoms. The methodology and insights from this study pave the way for future research in NLP and biomedical informatics, with implications for a wide range of applications beyond AE surveillance, such as drug discovery and clinical decision support.

## Figures and Tables

**Table 1:** Number of ‘induce’ relationships for each set

	Number of relations
**Train**	647
**Validation**	65
**Test**	59

## Data Availability

The datasets generated during and/or analyzed during the current study are available from the corresponding author upon reasonable request.
